# The Role of GCN2 Kinase in Mediating the Effects of Amino Acids on Longevity and Feeding Behaviour in *Drosophila*

**DOI:** 10.3389/fragi.2022.944466

**Published:** 2022-06-21

**Authors:** Anchal Srivastava, Jiongming Lu, Dennis Said Gadalla, Oliver Hendrich, Sebastian Grönke, Linda Partridge

**Affiliations:** ^1^ Max Planck Institute for Biology of Ageing, Cologne, Germany; ^2^ Department of Genetics, Evolution and Environment, Institute of Healthy Ageing, University College London, London, United Kingdom

**Keywords:** *Drosophila*, GCN2, ATF4, feeding behaviour, longevity, amino acid deficiency

## Abstract

Restriction of amino acids in the diet can extend lifespan in diverse species ranging from flies to mammals. However, the role of individual amino acids and the underlying molecular mechanisms are only partially understood. The evolutionarily conserved serine/threonine kinase General Control Nonderepressible 2 (GCN2) is a key sensor of amino acid deficiency and has been implicated in the response of lifespan to dietary restriction (DR). Here, we generated a novel *Drosophila GCN2* null mutant and analyzed its response to individual amino acid deficiency. We show that GCN2 function is essential for fly development, longevity and feeding behaviour under long-term, but not short-term, deprivation of all individual essential amino acids (EAAs) except for methionine. GCN2 mutants were longer-lived than control flies and showed normal feeding behaviour under methionine restriction. Thus, in flies at least two systems regulate these responses to amino acid deprivation. Methionine deprivation acts *via* a GCN2-independent mechanism, while all other EAA are sensed by GCN2. Combined deficiency of methionine and a second EAA blocked the response of GCN2 mutants to methionine, suggesting that these two pathways are interconnected. Wild type flies showed a short-term rejection of food lacking individual EAA, followed by a long-term compensatory increase in food uptake. GCN2 mutants also showed a short-term rejection of food deprived of individual EAA, but were unable to mount the compensatory long-term increase in food uptake. Over-expression of the downstream transcription factor ATF4 partially rescued the response of feeding behaviour in GCN2 mutants to amino acid deficiency. Phenotypes of GCN2 mutants induced by leucine and tryptophan, but not isoleucine, deficiency were partially rescued by ATF4 over-expression. The exact function of GCN2 as an amino acid sensor *in vivo* and the downstream action of its transcription factor effector ATF4 are thus context-specific with respect to the EAA involved.

## Introduction

Amino acids are key nutrients in mediating the beneficial effects of dietary restriction on healthy ageing (Grandison et al., 2009; Solon-Biet et al., 2014). Restriction of specific amino acids has been shown to extend lifespan and health span in diverse species. Methionine restriction increases longevity in flies and rodents (Orentreich et al., 1993; Miller et al., 2005; Lee et al., 2014). Restriction of the branched-chain amino acids (BCAA) leucine, isoleucine, and valine, extends lifespan in flies and leads to improved metabolic health, reduced frailty and lifespan extension in mice (Fontana et al., 2016; Juricic et al., 2020; Richardson et al., 2021). Despite the evident effects of amino acid restriction in healthy ageing, the underlying molecular mechanisms are still only partly understood.

Amino-acid-sensing pathways play key roles in the responses to dietary amino acid restriction (Babygirija and Lamming, 2021; Green et al., 2022). The Target of Rapamycin complex 1 (TORC1) directly senses amino acid availability through specific amino acid sensor proteins such as Sestrin (Zoncu et al., 2011; Wolfson et al., 2016). Inhibition of TORC1 by the drug rapamycin or *via* activation of the amino acid sensor Sestrin can extend lifespan (Bjedov et al., 2010; Miller et al., 2014; Lu et al., 2021). The evolutionary conserved eIF2α kinase General Control Nondepressible 2 (GCN2) indirectly senses amino acid deficiency (Gallinetti et al., 2013) and is activated by binding to uncharged t-RNAs (Wek et al., 1995; Dong et al., 2000). Activated GCN2 then phosphorylates its main substrate eIF2α, resulting in general inhibition of global translation and a concomitant activation of selective translation of mRNAs with specific regulatory elements in their 5′ UTRs (Wek et al., 1995; Qiu et al., 2001). These mRNAs include transcription factors such as GCN4 in yeast (Hinnebusch, 1997), and activating transcription factor 4 (ATF4) in mammals (Harding et al., 2003). ATF4 then regulates specific target genes to relieve the cell from amino acid starvation (Harding et al., 2003; Wek et al., 2006; Sikalidis et al., 2011). GCN2 has been suggested to be important for DR-mediated longevity in worms and flies (Rousakis et al., 2013; Kang et al., 2017), and regulates beneficial effects of amino acid restriction in mice (Longchamp et al., 2018). However, the *in vivo* functions of GCN2 under deficiency of individual amino acids are largely unknown.

Higher organisms are able to regulate their feeding behaviour based on the quality and quantity of dietary amino acids (Gietzen et al., 2007). In flies and rodents, essential amino acids (EAA) are one of the most important determinants for food choice (Koehnle et al., 2003; Leitao-Goncalves et al., 2017). Studies in *Drosophila* larvae and mice have indicated that rapid rejection of EAA-deficient diets is due to GCN2-mediated neuronal sensing of amino acids (Hao et al., 2005; Maurin et al., 2005). However, a more recent study showed that mice were not able to rapidly sense and reject leucine- or threonine-deficient diets and that GCN2 was not activated in the brain under EAA-deficient diets (Leib and Knight, 2015). The roles of GCN2 in mediating feeding behaviour under amino acid deficiency are thus still under debate.

Here, we generated a novel *Drosophila GCN2* null mutant and analyzed its effect on the responses to individual amino acid deficiency. We show that GCN2 function is essential for fly development, longevity and feeding behaviour under long-term, but not short-term, deprivation of individual EAAs, with the exception of methionine. These results suggest that in *Drosophila* the organismal response to EAA deprivation is regulated by at least two systems. While methionine deprivation acts *via* a GCN2-independent mechanism, all other EAA are sensed by GCN2. Combined deficiency of methionine and a second EAA blocked the response of GCN2 mutants to methionine, suggesting that these two pathways are interconnected. We further show that feeding behaviour of the GCN2 mutant was rescued by over-expression of the downstream transcription factor ATF4, but in an EAA-dependent manner. The function of GCN2 as an amino acid sensor *in vivo* and its downstream signalling is thus context-specific depending on the EAA involved.

## Materials and Methods

### Fly Husbandry

All fly stocks were maintained in culture bottles at 25°C on a 12:12 h light:dark cycle at constant humidity (65%) on a standard SYA [10% (w/v) brewer’s yeast, 5% (w/v) sucrose, and 1.5% (w/v) agar] food (Bass et al., 2007). All experimental flies were reared at standard larval density on SYA media. Newly emerged adults were collected over a period of 24 h and transferred to fresh food and allowed to mate for ∼48 h (once-mated). Subsequently, male or female flies were selected under brief CO_2_ anaesthesia and transferred to experimental vials. If not indicated otherwise, female flies were used. The UAS-ATF4 line was received from Fly ORF (accession #F000106), Zürich, Switzerland. All fly lines were backcrossed for at least six generations into the white Dahomey (*w* ^*Dah*^) background.

### Generation of *Gcn2* Null Mutants

The *Gcn2* ^*1*^ null mutant was generated by ends-out homologous recombination according to the methods described in (Gong and Golic, 2003; Gong and Golic, 2004; Huang et al., 2009). In the *Gcn2* ^*1*^ mutant, the complete ORF of the *Gcn2* gene was replaced by a *white* ^*hs*^ *marker* gene. Donor constructs used for targeting *Gcn2* were generated by amplifying approximately 4 kb of flanking sequences of the corresponding region of the *Gcn2* gene and subsequently cloned into the pGX attP vector (Huang et al., 2009). Long-range PCR was done using Takara LA Taq (Clontech, Cat# RR002A) and primer combinations SOL310/311 and SOL312/313 for the 5′ and 3′ arm, respectively, of the *Gcn2* ^*1*^ donor construct. BAC clones CH321-12O13 and RP98-2GL6 (BACPAC Resource Center, United States) were used as PCR template for the 5′ and 3′ arms, respectively. *Gcn2* donor constructs were full-length sequenced before the generation of transgenic fly lines by P-element-mediated germline transformation using the Best Gene *Drosophila* Embryo Injection Services (Chino Hills, California, United States). Ends-out homologous recombination was done following the rapid targeting scheme (Gong and Golic, 2003) and homologous recombination events were identified by genetic mapping of the *white* ^*hs*^ *marker* gene and subsequent PCR analysis using primers SOL365/366 (Supplementary Table S1).

### PCR Genotyping

Genomic DNA was isolated from adult flies using the DNeasy kit (Qiagen, Cat# 69504). DNA fragments were amplified with primer pairs (SOL365/SOL366), (SOL636/SOL637), (SOL639/SOL640), and (SOL336/SOL315) using HotStar Taq Plus master mix (Qiagen, Cat# 203603) according to the manufacturer’s instructions. PCR was performed for 35 cycles (30 s 94°C, 30 s 55°C, and 60 s 72°C) preceded by 5 min of initial denaturation at 95°C and followed by 10 min final elongation at 72°C. Gel electrophoresis was done with TAE buffered 1% agarose gels. Primer sequences are listed in Supplementary Table S1.

### RNA Extraction and Northern Blotting

Total RNA was isolated using Trizol-Chloroform (Thermo Fisher Scientific, Cat# 15596026) according to the manufacturer’s instructions. mRNA was extracted using the Dynabeads mRNA Direct Purification Kit (Thermo Fisher Scientific, Cat# 61012) from Trizol-Chloroform isolated total RNA. Northern blots were performed using the Northern Max Kit (Thermo Fisher Scientific, Cat# AM1940) according to the manufacturer’s instructions using 2 μg mRNA per lane. Northern blot probes were generated as follows: DNA fragments corresponding to the 5′ region (primers: SOL368/403) and 3′ region (SOL404/405) of *Gcn2* and the *CG31002* (SOL326/402) and *CG11337* (SOL336/315) genes were PCR amplified using BAC CH321-12O13 (BacPac Resource Center) as a template and cloned into the *pCRII* vector using the TOPO TA Cloning Kit (Thermo Fisher Scientific, Cat# K450001). Probes were produced by digesting 2 μg of the plasmid containing the fragments described above with EcoRI, and 25 ng of each probe was randomly primed using Klenow Fragment (5 U) and 10 μCi [α32P]-dCTP (3,000 Ci/mmol) per reaction. Overnight hybridization was carried out at 42°C with 10^6^ cpm/ml probe. For normalization, blots were re-hybridized with a probe detecting ribosomal protein *RpL32* transcripts. Primer sequences are listed in Supplementary Table S1.

### Protein Extraction and Immunoblotting

For protein extraction, 10-day old female flies were snap-frozen in liquid nitrogen. Flies were homogenized in RIPA-1% SDS buffer supplemented with Complete mini protease inhibitor without EDTA (Roche, Cat# 11836170001) and PhosStop phosphatase inhibitors (Roche, Cat# 04906837001). Protein content was determined by BCA assay (Pierce, Cat# 23225). 20 μg total protein was mixed with 4x SDS loading buffer (950 μl 4x Laemmli sample buffer with 50 μl β-mercaptoethanol as a reducing agent) and boiled for 5 min at 95°C. Proteins were separated on 12% SDS-PAGE gels (BioRad) and transferred to PVDF membranes (GE Healthcare, Cat# GE10600023). After blocking nonspecific binding with 5% nonfat dry milk powder in TBST (0.1% Tween20), blots were incubated with primary antibodies (p-eIF2α—1:1,000 dilution, Abcam, ab32157; t-eIF2α—1:5,000 dilution; β-Actin—1:10000 dilution, Abcam, ab8224), washed with TBST and incubated with HRP conjugated anti-mouse or anti-rabbit secondary antibodies (1:10,000 dilution, Invitrogen G-21234, G21040). The t-eIF2α antibody was custom-made by Eurogentec as described in (Andersen and Leevers, 2007). Detection was done by chemiluminescence using ECL kits (Pierce). Bands were quantified using ImageJ (Scion Software).

### Preparation of Chemical Diet

Holidic media, Yaa, and HUNTaa, were prepared according to (Piper et al., 2014). Briefly, sucrose, agar, amino acids with low solubility (L-isoleucine, L-leucine, and L-tyrosine), metal ions and cholesterol were combined with Milli-Q water and autoclaved at 120°C under constant stirring for 15 min in a Mediaclave 10 media preparator (Integra Biosciences). After autoclaving, sterile-filtered stock solutions of buffer, essential and non-essential amino acids, vitamins, nucleosides, choline, inositol, and preservatives were added. For lifespan assays with RU486 (Mifepristone, Sigma), the drug was dissolved in ethanol and added to the Yaa food to a final concentration of 100 μM or 50 μM. Control food contained the same volume of ethanol without the addition of RU486. Diets with modified amino acid content were prepared similar to Yaa or HUNTaa media, by only changing the content of the specified amino acid in the EAAs stock solution. Leucine and isoleucine were added as solid powder directly to the food and adjusted accordingly (Supplementary Tables S2, S3).

### Viability and Body Weight

For viability assays, flies were allowed to lay eggs for a period of 4–5 h on grape juice plates. 250 eggs per genotype and diet were picked and transferred to vials containing experimental food at a density of 25 eggs per vial and kept at 25°C. Newly eclosed adult flies were scored at regular intervals. At the end of the experiment, egg-to-adult viability was calculated as the percentage of the total number of eclosed adult flies per genotype and diet. For body weight determination, flies were briefly anesthetized on ice and weighed in pairs on a ME235S analysis balance (Sartorius).

### Lifespan Assay and Fecundity

For lifespan experiments, 100–200 once-mated male or female flies per genotype and diet, were maintained at a density of 10 flies per vial containing different experimental diets. The sorting day was classified as day 0 of the lifespan experiment. Flies were transferred to fresh vials every 2–3 days and the number of dead flies was scored on the day of transfer. For fecundity assays, eggs were collected over 15–20 h periods at several timepoints during the first 3–4 weeks of lifespan experiments. Numbers of eggs laid per vial at each time point was scored using a hand counter. Values are expressed as cumulative eggs laid per female fly.

### Proboscis-Extension Assay

For feeding assay, newly emerged adults flies were allowed to mate on fresh Yaa medium for 48 h. Subsequently, female flies were sorted and kept at a density of five per vial on Yaa medium for a day. Flies were then transferred to vials containing experimental diets. The next day in the morning (2 h after the lights were switched on), feeding rates were measured using a proboscis-extension assay in undisturbed conditions as previously described (Wong et al., 2009). Flies were observed for a period of 90 min/day, with feeding events recorded every 10 min. Feeding data is expressed as a proportion by experimental group: sum of scored feeding events/total number of feeding opportunities, where the total number of feeding opportunities = number of flies in vial × number of vials in the group × number of observations.

### Blue-Dye Feeding Assay

Blue-dye feeding assay was done as described in (Wong et al., 2009). Briefly, flies preconditioned on single EAA-deficient diets were transferred to the same single EAA-deficient-diet containing 2.5% blue dye (w/w; FD&C Blue No.1, Sigma). Feeding was observed for 2 h and flies were then flash-frozen in liquid nitrogen. The amount of blue dye was determined spectrophotometrically.

### Starvation Stress Assay

For starvation stress, 100 once-mated female flies per genotype and diet were allocated at a density of 20 flies per wide plastic vial. Flies were first kept on Yaa or single EAA deficient diets and transferred to fresh food vials every 2–3 days. After 7 days, flies were transferred to starvation medium (1% w/v agarose, Invitrogen, Cat# 16500-500). Dead flies were scored 3-4 times per day. For the starvation assay, with adult-onset of *Atf4* over-expression by inducible drivers, the entire set-up was the same except that the Yaa diet for mating period was supplemented with 100 μM and the experimental diets with 50 μM or 100 μM RU486 (Mifepristone) as indicated for each experiment.

### Triacylglyceride Measurement

Once-mated female flies were kept at a density of 20 per vial on different food for 7 days after which they were snap-frozen. Triacylglyceride (TAG) content quantification was performed according to Grönke et al. (2003). Briefly, frozen flies were homogenized in 1 ml of 0.05% Tween 20 followed by a heat-inactivation step for 5 min at 70°C and centrifugation at 14,000 rpm. 50 μl of the supernatant was incubated with 200 μl of Infinity™ Triglyceride Reagent (Thermo Fisher Scientific) at 37°C and absorbance was measured at 540 nm. Absolute TAG content was quantified using Triglyceride standards (Cayman Chemicals) and was normalized to the total protein content of the homogenate, determined by BCA assay (Pierce).

### RNA Seq Analysis

For RNA-Seq, female flies were kept for 3 days on Yaa, Yaa-M, Yaa-R, and Yaa-all medium after which they were snap-frozen in liquid nitrogen. Total RNA was extracted from head and thorax of 25 frozen flies per genotype and diet using Trizol-Chloroform (Thermo Fisher Scientific). RNA was treated using the RNase free DNase kit (Qiagen). 2 μg total RNA was used for library preperation using the polyA purification method. RNA sequencing was performed with three biological replicates on an Illumina HiSeq 2500 platform at the Max Planck Genome Center (Cologne, Germany) with 100 bp single-end reads and 35 million reads per sample. Raw reads were mapped to BDGP6.32 ENSEMBL build 103 using kallisto (v 0.46.2). Genes counts were quantified in the same step. Differential gene expression was calculated using DESeq2 (v 1.24.0) in R (v 3.6.3). Genes were annotated as expressed if they were contained in the DESeq2 output. Differentially regulated genes (adjusted *p* value < 0.05) were additionally subsetted into total genes, upregulated genes (log2 fold change > 0), and downregulated genes (log2 fold change < 0). Data were processed using custom python scripts and the following packages: numpy (v 1.12.0), scipy (v 1.7.1), and pandas (v 1.2.0). Results were visualized using the following packages: matplotlib (v 3.3.2), matplotlib-venn (v 0.11.5), and seaborn (v 0.11.1). Gene ontology enrichment analysis was performed using the DAVID API based function DAVIDenrich of the AGEpy python package (v 0.8.2) on the GOTERM_BP_FAT ontology to identify significantly enriched terms (Benjamini-Hochberg adjusted *p*-value < 0.05). As background, all expressed genes of a respective deprivation (for pairwise comparisons), or all expressed genes shared in all used gene sets (when comparing gene set overlaps) were used. Redundancy of significantly enriched GO terms (adjusted *p*-value < 0.05) was reduced using REVIGO (cutoff: “0.7”, valueType: “pvalue”, measure: “SIMREL”). GO terms were visualized using dotplot representation. 42 unique top GO terms from the top GO terms per condition (-R, -M, and -All), were sorted by adjusted *p*-value. Dotplots were clustered by the adjusted *p*-value using the clustermap algorithm (method: “average”, metric: “euclidean”) of the seaborn package.

### Statistical Analysis

For lifespan and starvation assays, data were recorded in Excel, and a log-rank test was performed. For small sample sizes (N < 10), data are presented as mean ± s.e.m. with individual data points shown. For samples sizes N ≥ 10 box-and-whisker plots were used with median, 25th and 75th percentiles, and Tukey whiskers indicated. Outliers are shown as open circles. Statistical methods are indicated in figure legends. Significance of overlap in venn diagram was calculated using one-tailed Fisher’s exact test. As background, all expressed genes of a respective deprivation (in pairwise comparisons), or all expressed genes (when comparing gene set overlaps) were used. *p* values less than 0.05 are considered to be statistically significant: ns, not significant; *p* < 0.05, *; *p* < 0.01, **; *p* < 0.001, ***; *p* < 0.0001, ****.

## Results

### GCN2 Function is Essential for Fly Development Under Restriction of Individual EAA

To explore the *in vivo* function of Gcn2 under amino acid deficiency, we generated a *Drosophila Gcn2* null mutant (*Gcn2* ^*1*^) by replacing the entire *Gcn2* ORF with a *white* ^*hs*^ marker gene using ends-out homologous recombination (Supplementary Figure S1A). PCR on genomic DNA using primer pairs targeting different regions of the *Gcn2* gene locus confirmed the absence of the gene in the *Gcn2* ^*1*^ mutant (Supplementary Figure S1B). Northern blot analysis showed no *Gcn2* RNA expression in the *Gcn2* ^*1*^ mutant (Supplementary Figure S1C), verifying that *Gcn2* ^*1*^ is a transcript null allele. Expression of the adjacent neighbour genes, *CG11337* and *CG31002*, was not affected by the insertion of the *white* ^*hs*^ marker gene (Supplementary Figure S1C), indicating that the mutation is specific to the *Gcn2* gene*.* Phosphorylation of eIF2α, the main substrate of GCN2 kinase, was significantly reduced in *Gcn2* ^*1*^ mutant flies compared to wild-type control flies (*w* ^*Dah*^) (Supplementary Figure S1D). Despite the low level of eIF2α, *Gcn2* ^*1*^ null mutant flies were homozygous viable and showed normal body weight when raised on a standard sugar-yeast-agar (SYA) diet (Supplementary Figures S1E,F).

**FIGURE 1:**
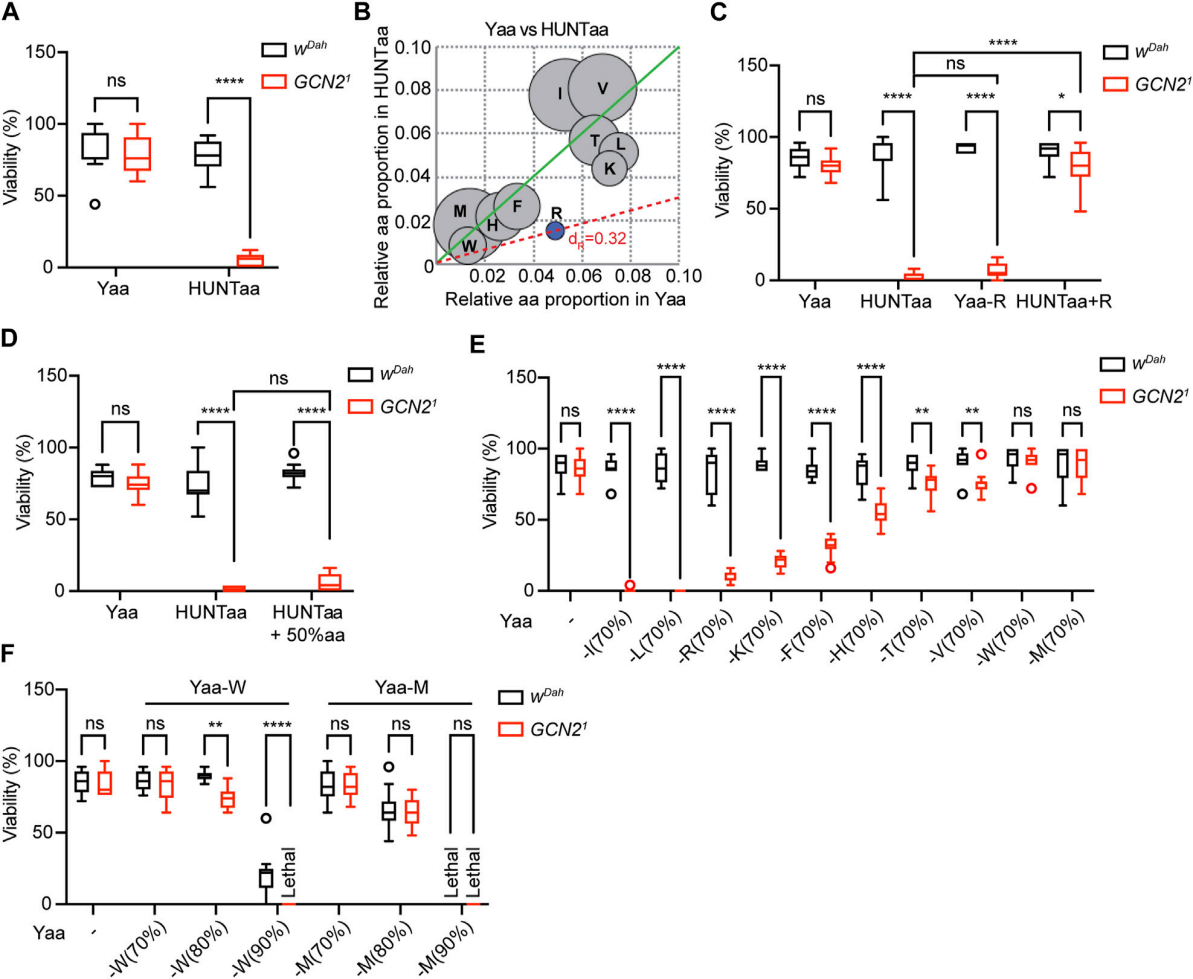
GCN2 function is essential for development under restriction of individual EAA except for methionine. **(A)** Egg-to-adult viability of *w* ^*Dah*^ control and *Gcn2* ^*1*^ mutants on chemically defined Yaa and HUNTaa diets. **(B)** Relative proportion of EAA in the Yaa diet compared to the HUNTaa diet. Arginine (R) highlighted in blue was identified as the most limiting EAA in HUNTaa. **(C)** Decreasing R in the Yaa diet by 70% (−R) reduced viability of *Gcn2* ^*1*^ mutants. Supplementation of R to the HUNTaa (+R) diet rescued viability of *Gcn2* ^*1*^ mutants. **(D)** Increasing the absolute amount of all aa by 50% in HUNTaa did not rescue viability of *Gcn2* ^*1*^ mutants. **(E)** Viability of *w* ^*Dah*^ control and *Gcn2* ^*1*^ mutants on Yaa diets with individual EAA restriction by 70%. **(F)** Viability of flies upon restriction of W and M by 80% and 90%. Viability (%) represents the percentage of eclosed adult flies per fly embryo. *n* = 10 vials with 25 embryos per vial. Median, 25th and75th percentiles, and Tukey whiskers are indicated. Two-way ANOVA followed by Bonferroni’s post-hoc test, *p* values were adjusted for multiple comparisons.

To assess the role of GCN2 in fly development under EAA deficiency, we employed chemically-defined (holidic) diets that allowed us to manipulate single amino acids. When raised on Yaa diet, which contains an amino acid composition based on yeast (Piper et al., 2014), *w* ^*Dah*^ controls and *Gcn2* ^*1*^ mutants displayed comparable egg-to-adult viability (Figure 1A), consistent with the result on the SYA diet (Supplementary Figure S1E). However, when subjected to a different holidic diet HUNTaa, which has a distinct amino acid ratio (Hunt, 1970; Piper et al., 2014), although *w* ^*Dah*^ controls showed normal viability, *Gcn2* ^*1*^ mutants showed dramatically reduced viability, and less than 10% of eggs developed into adult flies (Figure 1A). Therefore, GCN2 kinase is required for the development of flies on the HUNTaa diet.

To determine the reason behind this reduced developmental viability, we compared the amino acid composition of HUNTaa and Yaa (Figure 1B). HUNTaa diet contains ∼70% less arginine (R) than Yaa (Figure 1B). To test whether the reduced viability of *Gcn2* ^*1*^ mutants was attributable to R restriction in the HUNTaa diet, we first modified the Yaa diet by reducing the R content by 70% of its original level (Yaa-R, Figure 1C). Indeed, the viability of *Gcn2* ^*1*^ mutants on the Yaa-R diet was severely reduced and was comparable to the lethality observed on the HUNTaa diet (Figure 1C). YAA-R diet did not affect the viability of *w* ^*Dah*^ flies (Figure 1C). Next, we increased the R level in HUNTaa to the level of Yaa diet (HUNTaa + R). Interestingly, the viability of *Gcn2* ^*1*^ mutants was significantly rescued (Figure 1C), confirming that R restriction was causal for decreased viability of *Gcn2* ^*1*^ mutants on the HUNTaa diet. As the addition of R to the HUNTaa diet also changed its ratio to other amino acids, we then tested whether R restriction *per se* or changes in the amino acid ratio led to the reduced viability of *Gcn2* mutants. A modified HUNTaa diet that maintained the amino acid proportion but increased R level by scaling up the total amino acid content of HUNTaa diet to 150% (HUNTaa + 50% aa) was used. Interestingly, the viability of *Gcn2* ^*1*^ mutants was not rescued on this diet (Figure 1D), suggesting that the ratio of the limiting amino acid to the other amino acids, rather than the absolute amount of the limiting amino acid, in the diet is important for GCN2-dependent regulation of fly development.

We next investigated whether restriction of EAA other than arginine would also affect egg-to adult viability of GCN2 mutants (Figure 1E). 70% restriction of individual EAAs did not affect the viability of *w* ^*Dah*^ controls (Figure 1E). In contrast, viability of *Gcn2* ^*1*^ mutants was significantly reduced by 70% restriction of isoleucine (I, −100%), leucine (L, −100%), lysine (K, −76%), phenylalanine (F, −63%), histidine (H, −34%), threonine (T, −15%), and valine (V, −17%) (Figure 1E). 70% restriction of methionine (M) and tryptophan (W), did not significantly affect viability of *Gcn2* ^*1*^ mutants (Figure 1E). To test whether a stronger restriction of M and W would affect the viability of *Gcn2* ^*1*^ mutants, we reduced M and W levels by 80% and 90%, respectively (Figure 1F). Drop-down of W by 80% significantly decreased viability only of *Gcn2* ^*1*^ mutants. On 90% W, *Gcn2* ^*1*^ mutants failed to develop, while still 21% of control flies eclosed (Figure 1F), demonstrating that GCN2 function is also essential for development under W restriction. In contrast, titration of M levels to 80% and 90% affected *Gcn2* ^*1*^ mutants and *w* ^*Dah*^ controls to a similar extent, suggesting that GCN2 function is not essential for development under M restriction (Figure 1F). Taken together, our findings demonstrate that GCN2 is essential for fly development under the restriction of individual EAA, with a reduced role in W restriction and no role in M restriction.

### GCN2 Function is Essential for Adult Survival Under Restriction of Individual EAA Except Methionine

**FIGURE 2:**
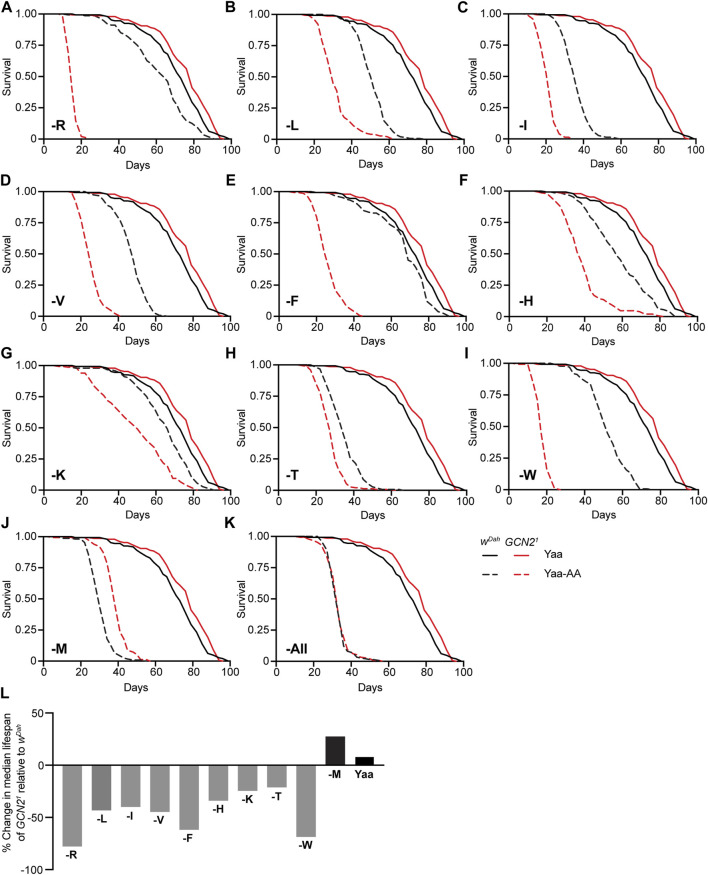
GCN2 function is essential for survival under deprivation of individual EAA except for methionine. **(A–K)** Survival of *w* ^*Dah*^ control and *Gcn2* ^*1*^ mutant female flies under full deprivation of individual EAA **(A–J)** and all amino acids (-All) **(K)**. *Gcn2* ^*1*^ mutants showed significantly increased lifespan compared to *w* ^*Dah*^ flies on the Yaa diet (*p* = 7.8 × 10^−3^, log-rank test). Survival of *Gcn2* ^*1*^ mutants was significantly reduced compared to *w* ^*Dah*^ on -R (*p* = 6.0 × 10^−73^) **(A)**, -L (*p* = 2.9 × 10^−41^) **(B)**, -I (*p* = 9.9 × 10^−63^) **(C)**, -V (*p* = 1.9 × 10^−64^) **(D)**, -F (*p* = 1.3 × 10^−67^) **(E)**, -H (*p* = 2.4 × 10^−25^) **(F)**, -K (*p* = 1.4 × 10^−12^) **(G)**, -T (*p* = 5.3 × 10^−15^) **(H)**, -W (*p* = 3.6 × 10^−72^) **(I)** diets, log-rank test. In contrast, *Gcn2* ^*1*^ mutants were significantly longer lived than *w* ^*Dah*^ control flies on M deprived medium (*p* = 4.4 × 10^−25^, log-rank test) **(J)**. Under deprivation of all amino acids **(K)**, *Gcn2* ^*1*^ mutants had a similar lifespan than *w* ^*Dah*^ flies (*p* = 0.66, log-rank test). Survival curves of *w* ^*Dah*^ and *Gcn2* ^*1*^ mutants on the Yaa diet are the same in **(A–K)**. **(L)** Summary of survival analysis represented as percentage change in median lifespan of *Gcn2* ^*1*^ mutants relative to *w* ^*Dah*^ controls on each diet. *n* = 150 flies for each condition of each genotype.

To examine the role of GCN2 in the response of adult flies to restriction of individual EAA, we measured survival of *w* ^*Dah*^ and *Gcn2* ^*1*^ female flies under full deprivation of individual EAA (Figures 2A–L). Restriction of individual EAA shortened lifespan of wild type females, although to different extents. Depletion of M, T, and I had the strongest effects, with a reduction in median lifespan by 60%, 54%, and 51%, respectively, compared to the Yaa control diet. Depletion of V, W, L, and H shortened median lifespan by 35%, 29%, 29%, and 22%, respectively, while reduction of R, K, and F had only minor effects in wild type females, with a decrease in median lifespan of 13%, 10%, and 6%, respectively (Figures 2A–J). Thus, lifespan reduction of wild type flies in response to EAA deficiency is dependent on the specific EAA restricted. Interestingly, *Gcn2* ^*1*^ mutants were slightly longer-lived than control flies on the Yaa control diet (Figure 2A). However, they had a significantly reduced median lifespan compared to control flies on nine of the ten single EAA-deficient diets (Figures 2A–I,L), including R (−78%), L (−43%), I (−40%), V (−45%), F (−62%), H (−34%), K (−25%), T (−21%), and W (−69%) (Figures 2A–I,L). Noteworthy, the lifespan response to EAA restriction differed between *Gcn2* ^*1*^ mutants and control flies. For example, R and F deficiency caused only minor effects in *w* ^*Dah*^ flies, while lifespan was severely reduced in *Gcn2* ^*1*^ mutants (Figures 2A,E). In contrast to the deprivation of all other EAA, *Gcn2* ^*1*^ mutants had an increased lifespan compared to *w* ^*Dah*^ control females under M deficiency (Figures 2J,L). Thus, GCN2 function is essential for survival under individual EAA deprivation with the exception of M.

Given its role under individual EAA restriction, we next asked whether GCN2 function was also essential for survival under complete deprivation of all EAA and non-EAA (-All). Complete deprivation of all amino acids strongly reduced survival of wild type females, which had a median lifespan of 30 days (Figure 2K). Interestingly, *Gcn2* ^*1*^ mutants showed the same lifespan as *w* ^*Dah*^ females in this condition, demonstrating that GCN2 function is not essential for survival under complete amino acid deprivation. Noteworthy, *Gcn2* ^*1*^ mutants were longer-lived under full amino acid starvation than under deprivation of specific EAA such as R and W, suggesting that full amino acid starvation might act *via* different mechanisms than deprivation of individual amino acids and is probably independent of GCN2 function.

Survival and fecundity are often correlated and egg laying is sensitive to dietary amino acid deficiency (Grandison et al., 2009). Thus, we measured fecundity of GCN2 mutants under individual EAA deprivation (Supplementary Figure S2A). As expected, egg laying was strongly reduced in *w* ^*Dah*^ females upon deprivation of all individual EAA and flies stopped producing eggs within 8 days on the deprived diet, suggesting that differences in egg production do not explain the observed differences in survival. GCN2 females laid as many eggs as control females on the Yaa control diet, but showed reduced fecundity compared to *w* ^*Dah*^ females on all EAA deprived diets including deprivation of M. In contrast, there was no difference in fecundity between *Gcn2* ^*1*^ and *w* ^*Dah*^ females under deprivation of all amino acids. Thus, like wild type flies, GCN2 mutants can downregulate their egg-production in response to EAA deficiency.

As dietary interventions often have sex-specific effects, we next measured survival of *w* ^*Dah*^ and *Gcn2* ^*1*^ male flies (Supplementary Figures S2B–D) under R, M, and -All deprivation. Consistent with the results obtained from females, *Gcn2* ^*1*^ mutant males were short-lived under R deprivation, showed an increased lifespan upon M deprivation and had the same lifespan compared to *w* ^*Dah*^ males under -All deprivation (Supplementary Figures S2B–D). Thus, GCN2 plays a similar role in males and females under EAA deprivation. In summary, our data show that GCN2 is an important mediator of fly survival under deprivation of individual EAA except for methionine, but it does not play a role in the response of adult survival to deprivation of all amino acids.

### GCN2 Regulates Food Intake in Response to Long-Term Deprivation of EAA

**FIGURE 3:**
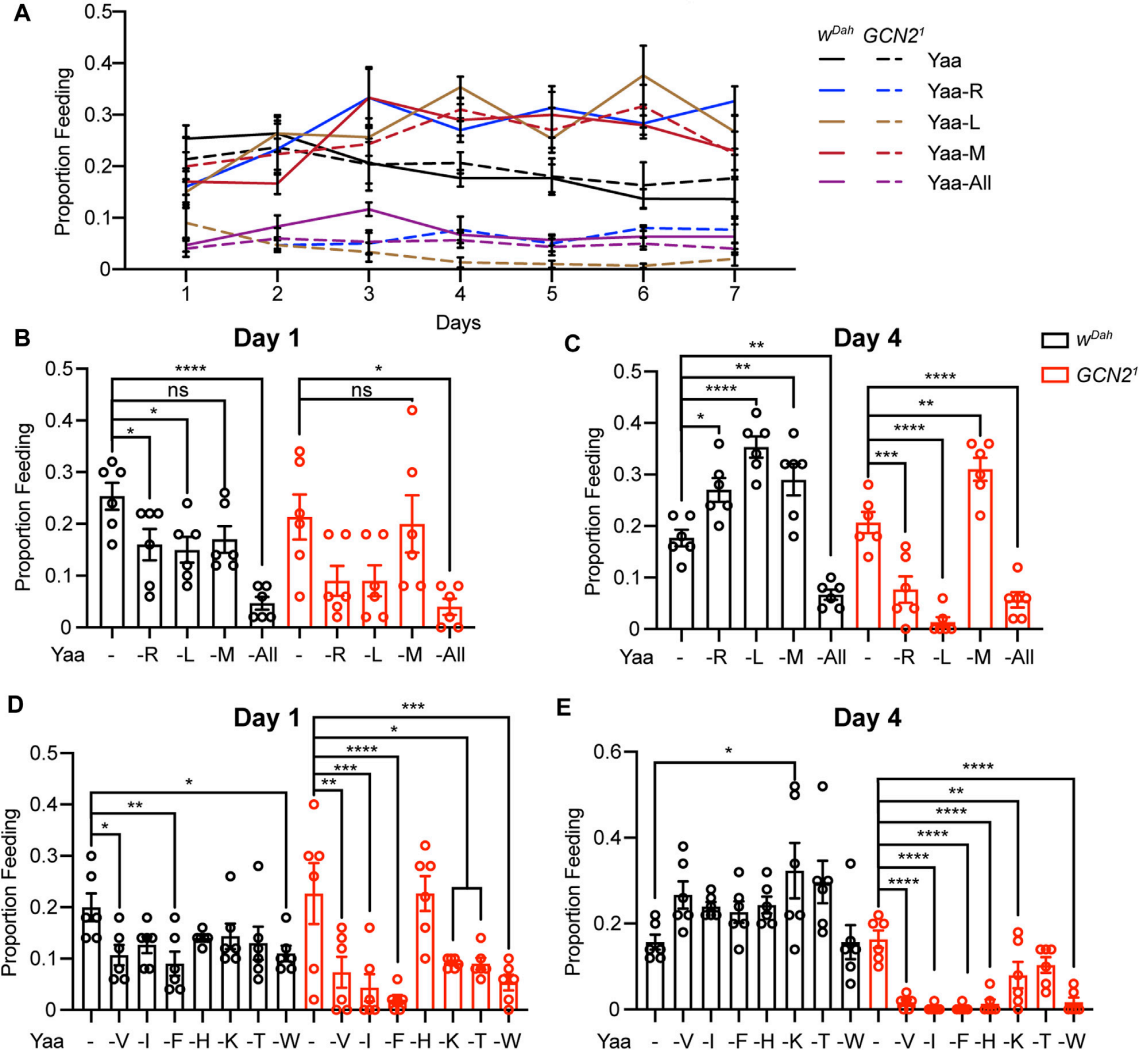
GCN2 regulates feeding behaviour under long-term but not short-term deprivation of individual EAA. **(A–C)** Feeding rates measured by proboscis extension assay of *w* ^*Dah*^ control and *Gcn2* ^*1*^ mutant females over a period of 7 days on Yaa, -L, -R, -M, and -All diets **(A)**. Feeding rates of *w* ^*Dah*^ controls and *Gcn2* ^*1*^ mutants on day 1 **(B)** and day 4 **(C)**. **(D,E)** Feeding rates of *w* ^*Dah*^ controls and *Gcn2* ^*1*^ mutants on V, I, F, H, K, T, and W deprived diets on day 1 **(D)** and day 4 **(E)**. *n* = 6 vials with five flies per vial. Data are present as mean ± s.e.m. One-way ANOVA followed by Dunnett’s multiple comparisons test, *p* values were adjusted for multiple comparisons.

GCN2 affects feeding behaviour in flies and mice (Hao et al., 2005; Maurin et al., 2005; Bjordal et al., 2014; Leib and Knight, 2015). In order to analyze how loss of GCN2 function affects feeding in response to deprivation of individual EAA, we performed proboscis extension assays (Wong et al., 2009) in adult *w* ^*Dah*^ and *Gcn2* ^*1*^ mutant females fed with individual EAA-deficient diets. To address both short-term and long-term responses, we measured feeding rates of the same flies successively over a period of 7 days on R, L, M, and -All deprived diets (Figure 3A). Short-term deprivation of R and L resulted in a significantly decreased food uptake of *w* ^*Dah*^ flies on day 1 compared to the Yaa control diet (Figure 3B), suggesting that flies are able to sense the lack of individual amino acids in their diet and avoid these deficient diets. The same tendency, although not significant, was also observed upon M deprivation (Figure 3B). The strongest reduction in food uptake was observed on the diet completely deficient of amino acids (Figure 3B). Interestingly, continued deprivation of R, L, and M resulted in increased feeding rates in *w* ^*Dah*^ flies, which were significantly different from the Yaa control diet on day 4 (Figure 3C), suggesting a long-term compensatory feeding response to the EAA-deficient diets. In contrast, *w* ^*Dah*^ flies did not increase their feeding rates on the -All diet (Figures 3B,C), which suggests that the presence of amino acids in the diet is essential to trigger compensatory feeding. *Gcn2* ^*1*^ mutants showed comparable feeding rates to *w* ^*Dah*^ flies on the Yaa control diet over the whole observation period (Figure 3A), indicating that GCN2 does not affect feeding rates under non-restrictive conditions. *Gcn2* ^*1*^ mutants also showed a tendency for reduced feeding upon short-term deprivation of R and L, suggesting that the mutants can sense the lack of these amino acids and initiate the avoidance behaviour. However, in contrast to wild type flies, GCN2 mutants were not able to increase their feeding rates upon prolonged deprivation of R and L (Figure 3A), indicated by the very low feeding rates on day 4 (Figure 3C). This suggests that GCN2 function is essential for the switch from avoidance to compensatory feeding upon EAA restriction. Interestingly, however, *Gcn2* ^*1*^ mutant flies showed increased feeding rates upon long-term restriction of M, demonstrating that, as for development and adult survival, GCN2 function is not essential for compensatory feeding in response to M deficiency. We next extended our analysis to the remaining EAA, and measured proboscis extension under short-term (day 1, Figure 3D) and long-term (day 4, Figure 3E) deprivation. Mostly consistent with the above results, restriction of individual EAA decreased feeding rates on day 1 for both *w* ^*Dah*^ and *Gcn2* ^*1*^ flies (Figure 3D). On day 4, *w* ^*Dah*^ flies showed a tendency for increased feeding upon deprivation of all EAA except for W, while GCN2 mutants showed strongly reduced feeding for all tested EAA (Figure 3E). Thus, GCN2 regulates long-term feeding behaviour in response to EAA deficiency.

Given that depletion of all amino acids did not induce long-term compensatory feeding in control or GCN2 mutant flies, we next tested whether restriction of all EAA or all Non-EAA (NEAA) would be sufficient to induce this response (Supplementary Figure S3A). However, deprivation of all EAA or all NEAA did not significantly affect feeding rates of control flies on day 4. In contrast, GCN2 mutants showed significantly reduced feeding rates under deprivation of EAA but surprisingly also under deprivation of NEAA (Supplementary Figure S3A). Thus, we asked next whether deprivation of individual NEAA might also affect long-term feeding behaviour of GCN2 mutants. As expected, feeding rates were not significantly changed in *w* ^*Dah*^ controls upon individual NEAA deprivation (Supplementary Figures S3B–D). *Gcn2* ^*1*^ mutant also showed no reduction in feeding rates upon deprivation of most NEAA except for asparagine (N), which strongly reduced feeding rates of mutant flies (Supplementary Figures S3B–D). To show that this was specifically due to reduced levels of N and not to overall changes in amino acid abundance, we fed flies with a diet containing EAA and only N, which was sufficient to rescue the feeding phenotype of *Gcn2* ^*1*^ (Supplementary Figure S3E). Thus, feeding of *Gcn2* ^*1*^ mutants is not only sensitive to deprivation of EAA but also deprivation of the NEAA asparagine.

Finally, as all our results were based on the proboscis extension assay, which is a good proxy for feeding but does not measure actual food uptake, we verified our core findings by performing a blue dye uptake assay (Wong et al., 2009). Consistent with the results from the proboscis extension assay, uptake of blue dye was increased in wild type flies upon long-term deprivation of L and I, while it was strongly suppressed in *Gcn2* ^*1*^ mutants on the same diets (Supplementary Figure S3F). Furthermore, we also confirmed increased feeding of GCN2 mutants upon long-term M restriction (Supplementary Figure S3F). Taken together, our results show that in adult flies GCN2 regulates long-term but not short-term feeding behaviour in response to EAA deficiency, with the exception of M deprivation, and that it plays a similar role in response to deprivation of the NEAA asparagine.

### GCN2 Function is Essential for Starvation Resistance Induced by Deprivation of EAA

**FIGURE 4:**
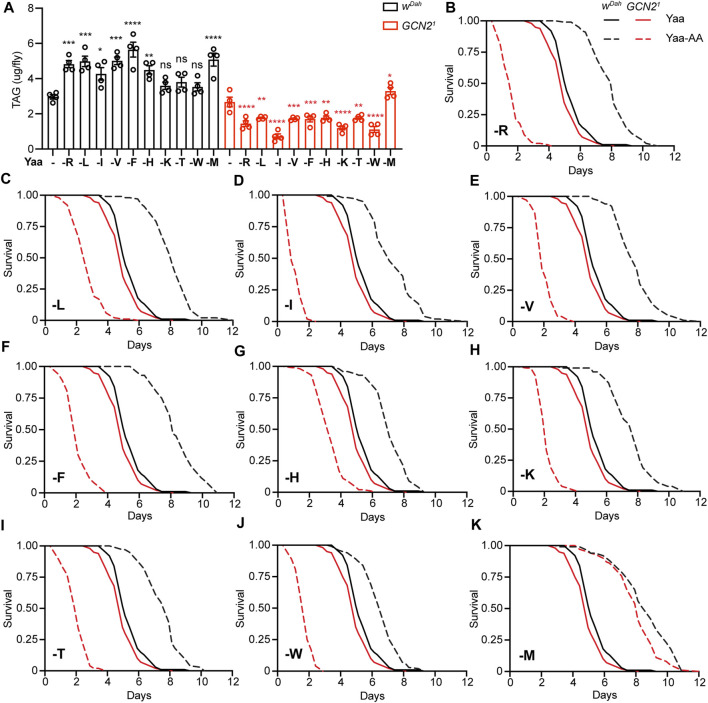
Lipid content and starvation resistance of GCN2 mutant flies in response to EAA deprivation. **(A)** TAG content per fly of *w* ^*Dah*^ and *Gcn2* ^*1*^ females upon deprivation of individual EAA. *n* = 4 replicates with five females per replicate. Data are present as mean ± s.e.m. One-way ANOVA followed by Dunnett’s multiple comparisons test, *p* values were adjusted for multiple comparisons. **(B–K)** Survival under full starvation of *w* ^*Dah*^ and *Gcn2* ^*1*^ females. Flies were fed for 7 days with the indicated EAA-deficient diets followed by full starvation. *Gcn2* ^*1*^ mutants preconditioned on the standard Yaa diet were slightly shorter-lived than *w* ^*Dah*^ control flies (*p* = 6.8 × 10^−3^, log-rank test). *w* ^*Dah*^ control flies preconditioned on EAA-deficient diets showed a significantly increased starvation when compared to the Yaa diet: -R (*p* = 2.3 × 10^−35^, log-rank test) **(B)**, -L (*p* = 3.4 × 10^−37^) **(C)**, -I (*p* = 5.2 × 10^−24^) **(D)**, -V (*p* = 5.2 × 10^−34^) **(E)**, -F (*p* = 7.7 × 10^−40^) **(F)**, -H (*p* = 1.9 × 10^−23^) **(G)**, -K (*p* = 2.6 × 10^−29^) **(H)**, -T (*p* = 3.3 × 10^−29^) **(I)**, -W (*p* = 2.3 × 10^−13^) **(J)**, -M (*p* = 1.1 × 10^−38^) **(K)**. *Gcn2* ^*1*^ mutants preconditioned on most EAA-deficient diets showed significantly decreased starvation resistance compared to the Yaa diet: -R (*p* = 8.9 × 10^−50^, log rank test) **(B)**, -L (*p* = 1.7 × 10^−36^) **(C)**, -I (*p* = 3.9 × 10^−52^) **(D)**, -V (*p* = 1.1 × 10^−49^) **(E)**, -F (*p* = 2.7 × 10^−48^) **(F)**, -H (*p* = 2.3 × 10^−23^) **(G)**, -K (*p* = 4.6 × 10^−49^) **(H)**, -T (*p* = 7.2 × 10^−49^) **(I)**, -W (*p* = 7.0 × 10^−51^) **(J)**. GCN2 mutants preconditioned with M-deprived food showed increased starvation resistance compared to the Yaa diet: -M (*p* = 3.4 × 10^−38^) **(K)** log-rank test. Survival curves of *w* ^*Dah*^ and *Gcn2* ^*1*^ on the Yaa control diet are the same in **(B–K)**. *n* = 100 flies per genotype and diet.

Food uptake is tightly connected to lipid metabolism and resistance to starvation (Gutierrez et al., 2007). Thus, we addressed whether triacylglyceride (TAG) levels and starvation resistance were affected in *w* ^*Dah*^ control and *Gcn2* ^*1*^ mutant females under deprivation of individual EAA (Figure 4). Flies were raised on individual EAA-deficient diets for 7 days and their TAG content was measured. Consistent with the increased feeding upon long-term EAA deprivation, *w* ^*Dah*^ control flies had a significantly higher TAG content under deprivation of all individual EAA compared to the Yaa control diet (Figure 4A). In contrast, total protein content of *w* ^*Dah*^ flies was reduced under individual EAA deprivation (Supplementary Figure S4A), which is possibly explained by reduced protein synthesis due to the limitation of EAA (Hinnebusch, 1994). *Gcn2* ^*1*^ mutants also showed reduced protein content under individual EAA deprivation, which was not different from *w* ^*Dah*^ flies on the respective diets (Supplementary Figure S4A). In contrast, TAG levels were reduced in *Gcn2* ^*1*^ mutants on all individual EAA-deficient diets compared to the Yaa control diet, except for M deprivation, where mutant flies showed an increased TAG content (Figure 4A).

As TAG storage and starvation resistance are often correlated, we measured survival of flies that were raised on individual EAA-deficient diets for 7 days and then subjected to full starvation (Figures 4B–K). *w* ^*Dah*^ flies preconditioned on individual EAA-deprived diets had significantly increased starvation resistance compared to flies raised on the Yaa control diet. In contrast, *Gcn2* ^*1*^ mutants showed significantly decreased survival under starvation for all individual EAA deprivations, except for M (Figures 4B–K), consistent with the feeding and TAG data. As feeding of GCN2 was also affected by restriction of all EAA, NEAA, or specifically N, we next measured starvation resistance of flies preconditioned on these diets (Supplementary Figures S4B,C). Total NEAA deprivation had similar effects to total EAA deprivation, increasing starvation resistance of *w* ^*Dah*^ controls and decreasing starvation resistance of *Gcn2* ^*1*^ mutants (Supplementary Figure S4B). Deprivation of individual NEAA did not affect starvation resistance of *w* ^*Dah*^ controls or *Gcn2* ^*1*^ mutants, except for N deprivation, under which *w* ^*Dah*^ and *Gcn2* ^*1*^ flies showed increased and decreased survival, respectively (Supplementary Figure S4C). Thus, overall we find a good correlation between feeding, TAG content and survival under starvation, suggesting that changes in feeding mostly underly the observed changes in TAG content and starvation resistance in response to EAA deprivation.

### Two Interconnected Mechanisms Regulate Long-Term Feeding Behaviour in Response to EAA Deficiency

**FIGURE 5:**
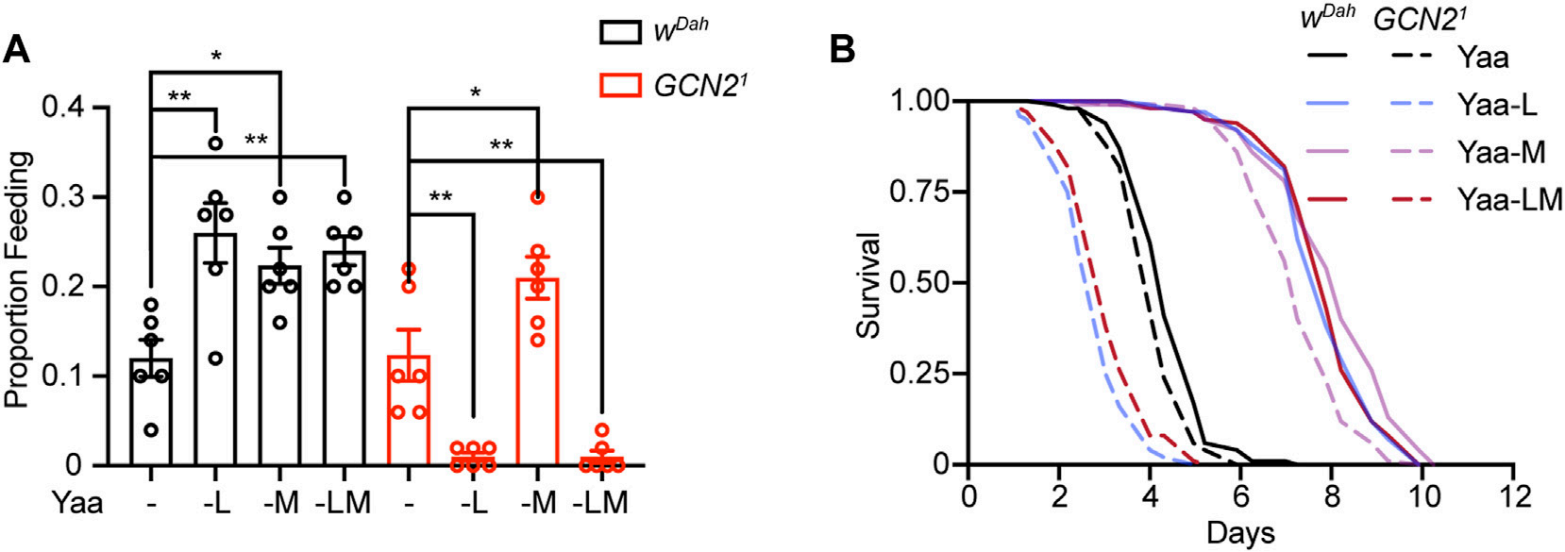
GCN2 mutants are not able to initiate compensatory feeding in response to combined deprivation of methionine and leucine. **(A)** Proboscis extension assay and **(B)** Starvation assay of *w* ^*Dah*^ controls and *Gcn2* ^*1*^ mutant females under combined deprivation of leucine and methionine. **(A)** Feeding rates of *w* ^*Dah*^ control flies were significantly increased but not different to each other upon long-term (day 4) deprivation of M, L, or LM. Feeding rates of *Gcn2* ^*1*^mutants were increased upon M deprivation, but strongly decreased in response to L and LM deprivation. *n* = 6 vials with five flies per vial. Data are present as mean ± s.e.m. One-way ANOVA followed by Dunnett’s multiple comparisons test, *p* values were adjusted for multiple comparisons. **(B)** Survival under full starvation of *w* ^*Dah*^ and *Gcn2* ^*1*^ females fed for 7-days with L, M, and LM deficient diets followed by full starvation. *w* ^*Dah*^ control flies were significantly starvation resistant upon L, M, and LM deprivation: (-L: *p* = 1.1 × 10^−45^, -M: *p* = 1.2 × 10^−44^, -LM: *p* = 9.5 × 10^−46^, log-rank test). *Gcn2* ^*1*^ mutants were significantly starvation resistant upon M deprivation, but showed decreased survial upon L and LM deprivation: (-L: *p* = 8.4 × 10^−23^, -M: *p* = 2.5 × 10^−47^, -LM: *p* = 8.4 × 10^−14^, log-rank test). *n* = 100 flies for each condition of each genotype.

Our data suggest that in flies long-term feeding behaviour in response to EAA deprivation is regulated by two mechanisms, one dependent on GCN2 and including all EAAs except M, and a GCN2 independent mechanism that is regulated by M. In order to address whether these mechanisms work independently in parallel or are interconnected, we subjected *w* ^*Dah*^ and *Gcn2* ^*1*^ mutant flies to a diet deficient for both M and L (-LM) and measured their feeding rate on day 4 (Figure 5A). If both pathways act in parallel, we would expect *Gcn2* ^*1*^ mutants to increase their feeding behaviour on the -LM diet, as they can sense the deficiency of M. However, in contrast to this hypothesis, feeding of *Gcn2* ^*1*^ mutants was strongly suppressed on the -LM diet, to a similar extent as under -L deprivation. In addition, feeding rates of *w* ^*Dah*^ control flies were increased to a similar extent upon -LM deprivation compared to individual deprivation of M and L (Figure 5A), suggesting that these systems do not act additively. Consistent with the feeding data, *w* ^*Dah*^ control flies showed a similar increase in starvation resistance upon -LM deprivation compared to the L or M deprived diets (Figure 5B). Furthermore, survival of *Gcn2* ^*1*^ mutants under starvation was decreased to a similar extent upon -LM deprivation and L deprivation (Figure 5B). In summary, these results suggest that the two mechanisms regulating feeding behaviour in response to long-term EAA deficiency are interconnected and that the lack of GCN2 function under EAA deficiency blocks activation of the M dependent branch.

### Transcriptional Profiling of GCN2 Dependent Expression Changes in Response to Amino Acid Deprivation

To gain further insights into the molecular changes associated with amino acid deprivation in *Drosophila* and the role GCN2 plays in this context, we performed transcriptomic profiling on representative amino acid deficient diets for *w* ^*Dah*^ control and *Gcn2* ^*1*^ mutant females. Flies were fed for 3 days with diets deprived for M, R, or all amino acids, corresponding to the time point when wild type flies switched their behaviour from avoidance to compensatory feeding. RNA was isolated from combined head and thorax samples to avoid profiling transcriptional changes associated with differences in egg production between females fed the standard and amino acid deficient diets (Supplementary Figure S2A).

**FIGURE 6:**
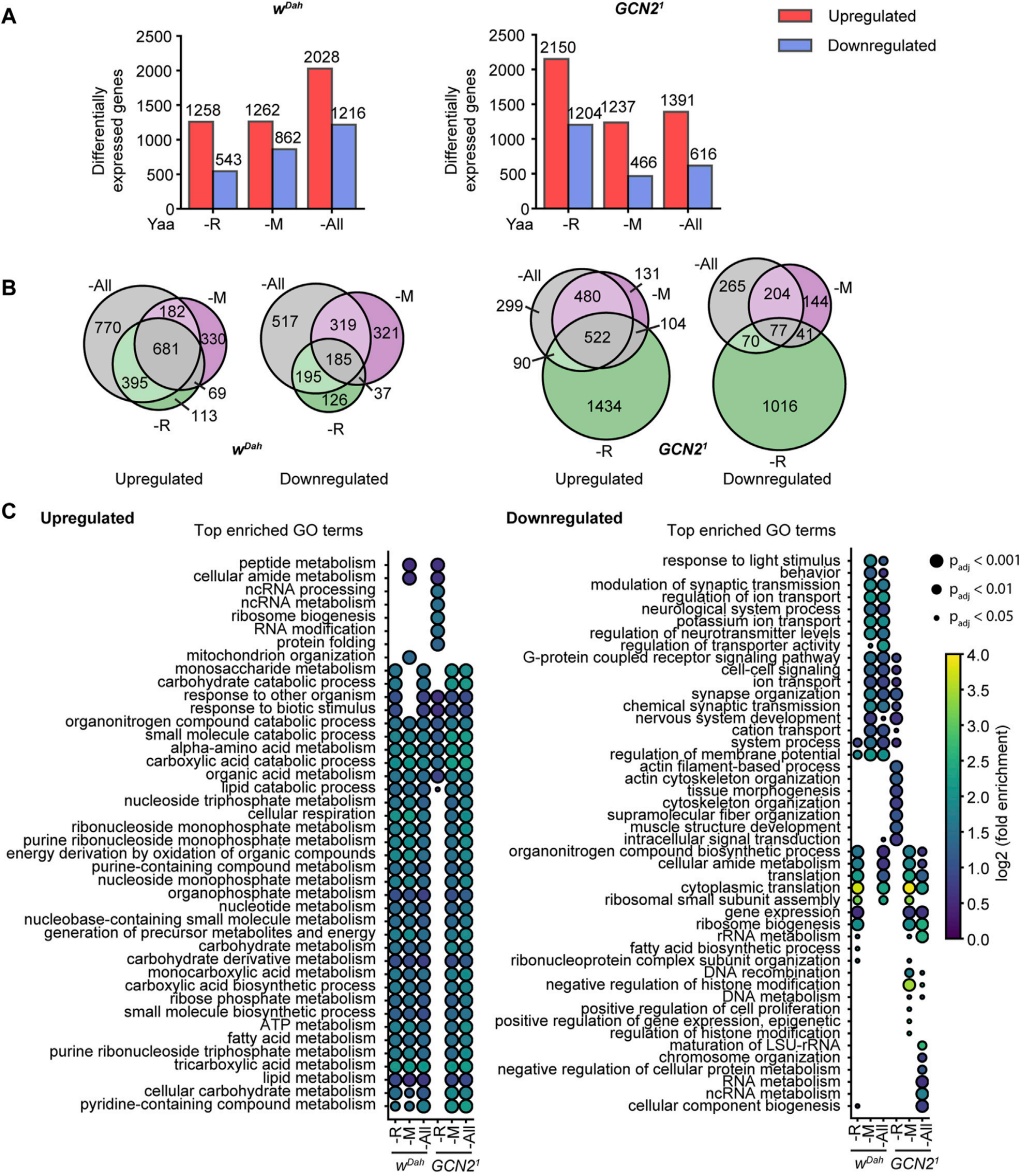
Transcriptional profiling of wild type and GCN2 mutants under amino acid deprivation. **(A)** Barplot representation of differentially expressed genes (DEGs) in *w* ^*Dah*^ control (left) and *Gcn2* ^*1*^ mutant females (right) under -R, -M, and -All deprivation relative to the Yaa control diet. The exact number of DEGs for each diet is indicated above the bar. **(B)** Venn representation of gene set overlaps of DEGs in the *w* ^*Dah*^ controls (left) and *GCN2* ^*1*^ mutants (right) under -R, -M, and -All deprivation. **(C)** Dotplot representation of significantly enriched top GO terms sorted by adjusted *p*-value of up and downregulated genes in each genotype and diet. *n* = 3 replicates per genotype and diet.

Three days of amino acid deprivation led to substantial differential gene expression changes in wild type flies with 1,801 (1,258/543), 2124 (1,262/862), and 3,244 (2028/1,216) genes significantly regulated (up/down) under R, M, and All deprivation, respectively (Figure 6A). 681 of upregulated genes and 185 of downregulated genes were shared between all amino acid deprived diets (Figure 6B), suggesting that they constitute a set of genes regulated in response to general amino acid deprivation. GO term analysis showed that genes associated with lipid metabolism, TCA cycle and nucleotide metabolism were among the shared processes upregulated by EAA deprivation (Figure 6C), consistent with the increased TAG content of wild type flies upon EAA restriction. In contrast, we also identified transcriptional profiles specific to individual EAA deprivations, with 113, 330, and 770 genes up and 126, 321, and 517 genes downregulated specifically upon deprivation of R, M, and All amino acids, respectively. For example, GO terms associated with mitochondrion organisation were significantly enriched exclusively in genes upregulated under M restriction, while terms associated with ribosome biogenesis were significantly enriched specifically in genes downregulated under R deprivation.

As in wild type flies, amino acid deprivation also led to substantial gene expression changes in *Gcn2* ^*1*^ mutants with 3,354 (2,150/1,204), 1,703 (1,237/466), and 2,007 (1,391/616) genes significantly regulated (up/down) under R, M, and All deprivation, respectively (Figure 6A). Compared to wild type flies, more genes were differentially regulated in *Gcn2* ^*1*^ mutants under R deprivation (Figure 6A) and the regulation of many of these genes was specific to R deficiency (Figure 6B). These flies also showed a very distinct GO term enrichment profile (Figure 6C). For example, they did not upregulate genes associated with lipid metabolism, consistent with the reduction in lipid storage and starvation sensitivity of *Gcn2* ^*1*^ mutants under R deprivation. There was a significant overlap of genes that were regulated in the same direction in both wild type and *Gcn2* ^*1*^ mutant flies on all diets (Supplementary Figure S5A), suggesting that loss-of GCN2 function does not block the general transcriptional programme in response to EAA deprivation. The biggest overlap was found under deprivation of all amino acids, with 1,195 up and 302 downregulated genes shared between *w* ^*Dah*^ and *Gcn2* ^*1*^ mutants, consistent with the hypothesis that GCN2 function is not essential for the response to deprivation of all amino acids.

Under EAA deprivation, GCN2 regulates transcription *via* translational induction of the transcription factor ATF4 (Harding et al., 2003). Thus, we next addressed whether ATF4 target genes might be enriched in our DEG gene sets. Therefore, we compared a list of 149 genes previously identified as being induced upon over-expression of ATF4 (Hunt et al., 2019) with our DEG sets (Supplementary Figure S5B). We found a significant overlap between potential ATF4 target genes and genes regulated in response to R and M deprivation in both *w* ^*Dah*^ and *Gcn2* ^*1*^ mutant flies (Supplementary Figure S5B), which suggest a role for ATF4 in the organismal response to these diets. However, the finding that the overlap was also observed in GCN2 mutants suggests that ATF4 target gene induction is independent of GCN2 function. To investigate this further, we next addressed whether ATF4 target genes were enriched in GCN2 dependent genes, i.e., genes that were regulated in wild type but not GCN2 mutants in response to EAA deprivation (Supplementary Figure S5C). Although we found some overlap, this overlap was not statistically significant. This suggests that factors other than ATF4 contribute to GCN2 dependent gene expression changes under these EAA deprivations. Interestingly, we found that induction of the known ATF4 target gene asparagine synthetase (ASNS) (Pathria et al., 2019), was dependent on GCN2 function specifically under M deprivation (Supplementary Figure S5D), suggesting that dependent on the type of EAA restriction ATF4 function is specifically regulated by GCN2. In summary, we identified both shared and distinct transcriptional profiles in response to EAA in flies. Some of these expression changes were dependent on GCN2, however, loss-of GCN2 function did not block the general transcriptional programme in response to EAA deprivation.

### GCN2-Dependent Feeding Behaviour in Response to EAA Deprivation Can be Rescued by ATF4 in an Amino-Acid-Specific Manner

**FIGURE 7:**
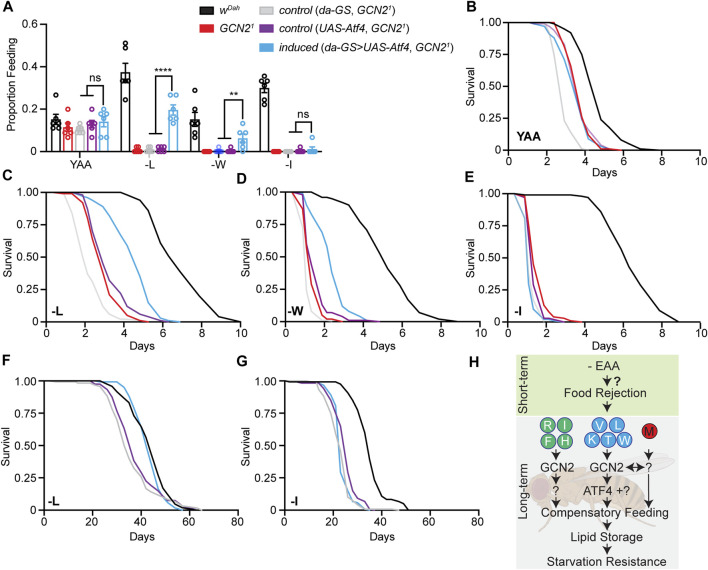
Over-expression of *Atf4* partially rescued feeding behaviour of *Gcn2* ^*1*^ mutants in an amino acid dependent manner. **(A)** Feeding rate of flies fed L, W, and I deprived diets for 7 days. Ubiquitous adult-specific overexpression of *Atf4* in *Gcn2* ^*1*^ mutant females rescued feeding behaviour under L and W but not I deprivation. *n* = 6 vials with five flies per vial. Data are present as mean ± s.e.m. One-way ANOVA followed by Dunnett’s multiple comparisons test; *p* values were adjusted for multiple comparisons. **(B–E)** Survival under full starvation of flies preconditioned on Yaa, -L, -W, and -I diets for 7 days. Ubiquitous adult-specific over-expression of *Atf4* in *Gcn2* ^*1*^ mutant females partially rescued starvation sensitivity on -L and -W but not on -I diets: -L: *p*_*da-GS*_ = 1.8 × 10^−34^, *p*_*UAS-Atf4*_ = 4.4 × 10^−13^; -W: *p*_*da-GS*_ = 2.1 × 10^−33^, *p*_*UAS-Atf4*_ = 7.6 × 10^−13^; -I: *p*_*da-GS*_ = 7.3 × 10^−12^, *p*_*UAS-Atf4*_ = 0.22, -I, *p*_*da-GS*_ = 0.32, *p*_*UAS-Atf4*_ = 4.9 × 10^−7^. log-rank test. *n* = 100 flies per diet and genotype. **(F,G)** Survival of flies under deprivation of -L **(F)** and -I **(G)**. Ubiquitous adult-specific over-expression of *Atf4* in *Gcn2* ^*1*^ mutant females rescued survival under L but not under I deprivation. -L: *p*_*da-GS*_ = 7.2 × 10^−6^, *p*_*UAS-Atf4*_ = 0.001. ATF4 overexpression in **(A–G)** was induced by 50 μM RU486 using the da-GS driver. **(H)** Schematic overview about the role of GCN2 under individual EAA deprivation. **(H)** was created with BioRender.com.

ATF4 is a main downstream effector of GCN2 and regulates expression and activity of genes involved in amino acid transport, assimilation, and metabolism under amino acid starvation (Harding et al., 2003; Kilberg et al., 2009). Surprisingly, we only identified a few ATF4 target genes in our transcriptomics analysis. Thus, we asked whether ATF4 is an important downstream effector of GCN2 dependent feeding behaviour in flies. We over-expressed ATF4 in *Gcn2* ^*1*^ mutant females and measured proboscis extension and starvation resistance in response to long-term deprivation of individual EAAs. To avoid developmental effects, we restricted expression of ATF4 specifically to the adult stage using the inducible Gene-Switch (GS) system (Roman et al., 2001). Expression of the *UAS-Atf4* transgene was induced 2 days prior to the flies being shifted to experimental diets using the ubiquitously expressed *da-GS* driver. *Gcn2* ^*1*^ mutants carrying only *da-GS* or *UAS-Atf4* constructs served as controls. We first measured feeding rates of flies in response to L, W, and I deprivation. Induction of ATF4 did not affect feeding of flies on the Yaa control diet (Figure 7A). Interestingly, however, ATF4 induction was able to partially rescue the reduced feeding rates of GCN2 mutants under L and W deprivation (Figure 7A). In contrast, feeding rates of GCN2 mutants were not rescued by ATF4 over-expression upon I deprivation (Figure 7A). Thus, ATF4 activation rescued GCN2 dependent feeding behaviour in an amino-acid-specific manner. As feeding behaviour and starvation resistance were closely correlated, we next validated this finding by measuring starvation resistance (Figures 7B–E). Consistent, with the feeding data, ATF4 over-expression partially rescued starvation resistance of GCN2 mutants under L and W, but not I, deficiency. Finally, we also tested whether over-expression of ATF4 can rescue the shortened lifespan of GCN2 mutants on two representative EAA diets, L and I deprivation, which showed partial rescue and no rescue in feeding and starvation resistance, respectively. Consistent with these results, over-expression of ATF4 fully rescued the lifespan of GCN2 mutants back to wild type levels upon L deprivation (Figure 7F) but was not able to rescue the reduced lifespan of GCN2 mutants under I deprivation (Figure 7G).

In the Gene-Switch system, transgene expression is induced when flies are exposed to the drug mifepristone (RU486). To check that the partial rescue was due to insufficient *Atf4* induction, we repeated the starvation experiment with a two-fold higher RU486 concentration. Furthermore, we extended our analysis to all individual EAA-deficient diets (Supplementary Figures S6A–J). Doubling the RU486 content only mildly increased the degree of rescue of starvation resistance of *Gcn2* ^*1*^ mutants under L, W, and I deprivation (Supplementary Figures S6A,B,J), suggesting that insufficient *Atf4* induction did not explain the partial rescue observed under these conditions. Furthermore, ATF4 over-expression partially rescued starvation resistance of GCN2 under deprivation of V, T, and K (Supplementary Figures S6C–E), while no or only a small rescue was observed under R, F, and H deprivation (Supplementary Figures S6F,H,I). Noteworthy, *Atf4* induction did not rescue starvation resistance of *Gcn2* ^*1*^ mutants under M deprivation, consistent with the hypothesis that M deprivation acts *via* a GCN2 independent mechanism. Taken together, overexpression of *Atf4* can partially rescue GCN2-dependent feeding behaviour and starvation resistance in an amino-acid-dependent manner. These results suggest that signalling downstream of GCN2 is amino acid specific and that factors other than ATF4 are also involved (Figure 7H).

## Discussion

Restriction of amino acids can extend healthspan and lifespan in animals ranging from flies to mammals (Grandison et al., 2009; Solon-Biet et al., 2014; Green et al., 2022). However, only a limited number of amino acids have been studied in this context (Babygirija and Lamming, 2021). A better understanding of the effects of individual amino acid restriction and the underlying molecular mechanisms will assist in developing novel nutrition-based interventions to improve health. Here we systematically analyzed the role of GCN2 kinase in development, survival and feeding behaviour under deprivation of individual amino acids in the fruit fly *Drosophila*. We show that GCN2 function is essential for the long-term response of the organism to deprivation of all essential amino acids, except for methionine.

Previous work on GCN2 in multicellular organisms has mostly been done using one or two prototype EAA-deficient diets (Maurin et al., 2005; Leib and Knight, 2015). We systematically examined the roles of GCN2 in regulating development, lifespan and starvation resistance in responses to individual amino acid deprivation, and found that GCN2-mediated responses vary in degree under deprivation of different EAA, suggesting that GCN2 is not comparably activated by each of the EAA. This is consistent with the results from culture cells that deprivation of individual EAAs increases phosphorylation of eIF2α, but to varying degrees (Tang et al., 2015) and deprivation of leucine and threonine result in the largest increase in p-eIF2α, whereas isoleucine, lysine, methionine, and tryptophan elicit the weakest responses (Palii et al., 2009). In addition, previous work with leucine deprivation (100%) and leucine restriction (85%) shows that the physiological responses to them are fundamentally different, thereby questioning the assumption that GCN2 is comparably activated by any degree of restriction of an EAA (Anthony et al., 2013). These findings indicate that both the EAA being deprived and the degree of restriction play important role in the activation of GCN2 kinase. Therefore, a precise way to dissect GCN2 functions is to study its responses under deprivation of individual amino acids. Interestingly, GCN2 seems to sense the ratio of the deprived amino acid to others, rather than the absolute amino acid levels, as the addition of 50% amino acid in the HUNTaa could not rescue the lethality caused by arginine limitation. Different dietary intervention regimes, such as protein restriction, general amino acid restriction, individual amino acid restriction, food dilution affect amino acid concentrations and ratios in different ways, and we hypothesize that GCN2 may play different roles mediating their effects.

Surprisingly, GCN2 mutants were slightly longer lived than control flies on the Yaa control diet. The Yaa diet is based on the amino acid composition of yeast (Piper et al., 2014) and addition of M to yeast can rescue the reduced fecundity under DR (Grandison et al., 2009), indicating that M is limiting in the yeast based diet. GCN2 mutants were longer-lived under full deprivation of M. Thus, the increased lifespan of GCN2 mutants on the control diet might be explained by partial M restriction, but could also have other currently unknown reasons.

We previously showed that 85% restriction of the three BCAA (V, L, and I) or of three other EAA (T, H, and K) extends lifespan in flies (Juricic et al., 2020). Combined restriction of BCAA or THK also caused short-term food avoidance followed by long-term compensatory feeding associated with increased lipid storage and starvation resistance (Juricic et al., 2020), consistent with our findings on full deprivation of individual EAA. Thus, in flies partial restriction of BCAA affects feeding behaviour in a similar manner as full deprivation of individual EAA. In contrast, full deprivation of individual EAA shortened lifespan. Restriction of BCAA and THK resulted in reduced TOR signalling (Juricic et al., 2020) and GCN2 has been implicated in TOR-mediated longevity in *Caenorhabditis elegans* (Rousakis et al., 2013). Therefore, in future it will be interesting to address whether GCN2 function is essential for lifespan extension upon BCAA or THK restriction.

Higher organisms actively regulate feeding behaviour based on the quality and quantity of amino acids in the diet. Mice fed with diets restricted for M and L (Lees et al., 2017) or T (Yap et al., 2020) for 8 weeks showed increased food uptake, consistent with our finding in flies that individual EAA deprivation induced compensatory feeding. Interestingly, while T restriction also increased fat content in mice, similar to our observation in flies (Yap et al., 2020), M and L restriction reduced fat content (Lees et al., 2017). It is currently not clear whether these differences are due to different functions of these EAA or due to other factors like differences in diet composition between studies. However, the evolutionarily conservation of increased food uptake in response to EAA restriction between flies and mice, suggest that also the molecular pathways underlying these effects are conserved. GCN2 is involved in the adaptive response of food intake in response to EAA deprivation (Hao et al., 2005; Maurin et al., 2005; Bjordal et al., 2014). In mice, GCN2 activation leads to rapid rejection of a leucine or threonine deficient diet within an hour of feeding (Maurin et al., 2005). Similarly, fly larvae rapidly reject a sugar/corn-based diet deficient in tryptophan and lysine (Bjordal et al., 2014). However, another study in mice reported that, under long-term leucine deprivation (7 and 17 days), both wild-type and *Gcn2*-deficient mice show reduced food-intake (Guo and Cavener, 2007). Furthermore, a more recent study has called the rapid rejection of EAA-deficient diets into question, and showed that mice cannot rapidly sense and reject leucine or threonine deficient diets (Leib and Knight, 2015). Thus, the role of GCN2 in this context is still under controversy. We performed a comprehensive analysis of GCN2-mediated food intake in response to short-term and long-term deprivation of individual EAA in adult *Drosophila* females and the main finding were: 1) short-term (1 day) deprivation of individual EAAs triggers a GCN2-independent aversion response towards EAA-deficient diets, 2) long-term (3–7 days) single deprivation of individual EAA except for methionine induces a GCN2-dependent compensatory feeding response in wild type flies, 3) full amino acid deprivation does not cause a compensatory feeding response and is sensed by a GCN2-independent mechanism, and 4) long-term methionine deprivation is sensed by a GCN2 independent mechanism, suggesting the presence of at least two independent amino acid sensing systems. In contrast to fly larvae (Bjordal et al., 2014), we did not observe a short-term GCN2-dependent food rejection in adult flies. There are several possible explanations for the observed discrepancy, including differences between larval and adult behaviour, different food composition and differences in the measurement period (few minutes vs. 24 h). Short-term deprivation of EAAs is sensed by a currently unknown mechanism that elicits a rejection response in adult flies. However, when EAA deprivation continues over a longer period, GCN2 kinase is activated and might receive feedback signals from the short-term sensing pathway to trigger a transcriptional response, which induces compensatory feeding in flies in order to cope with the nutritional stress. We propose that feeding behaviour in response to an EAA-deficient diet is highly dependent on the duration of the amino acid starvation and is mediated, at least in part, by GCN2 kinase.

Our results suggest that methionine deprivation is sensed by a GCN2-independent mechanism. These findings are in line with a recent study in mice that showed that GCN2 is not required for methionine-restriction-dependent physiological responses including increased food intake, induction of hepatic FGF21, increased energy expenditure, or enhancement of insulin sensitivity (Wanders et al., 2016). Methionine participates in multiple cellular metabolic pathways, including the salvage pathway, the SAM recycling pathway, the transsulfuration pathway, polyamine synthesis, and creatine biosynthesis (Drabkin and RajBhandary, 1998; Stipanuk and Ueki, 2011; Tang et al., 2015) and hence methionine deprivation could act through multiple molecular mechanisms. Along the same lines, a comprehensive microarray analysis in a mammalian cell line demonstrated that, unlike deprivation of other EAAs, methionine deprivation triggers a unique and dramatic gene expression response through a reduction of both histone methylation and ornithine-mediated signaling (Tang et al., 2015). A recent study showed that methionine starvation had no significant effect on phosphorylation of eIF2α (Mazor et al., 2018), indicating that methionine deprivation may not regulate GCN2. Interestingly, general translation was drastically decreased under methionine starvation, consistent with strong reduction in adult survival we observed (Mazor et al., 2018). The exact molecular mechanisms should be further investigated.

Feeding behaviour of GCN2 mutants was not only affected by deprivation of EAAs but also by deficiency of NEAA. More specifically, deprivation of asparagine (N), but not of other NEAA, reduced feeding and decreased starvation resistance of GCN2 mutants. Furthermore, NEAA and N deprivation increased starvation resistance of wild type flies, but not their feeding behaviour. This suggests that mechanisms other than increased food uptake underlie the increased starvation resistance upon N deprivation. The observed phenotypes specifically upon N deprivation might indicate that flies only have a limited production capacity for this NEAA. In mammalian cell culture cells, N becomes an EAA under deprivation of glutamine (Pavlova et al., 2018). However, deprivation of N alone in the presence of external glutamine was sufficient to affect starvation resistance of flies, suggesting that either the level of glutamine in the Yaa control diet is limiting or that other mechanisms are at play. N has also been shown to act as an amino acid exchange factor, whereby intracellular N levels regulate the uptake of other amino acids, especially, serine, arginine and histidine (Krall et al., 2016). Thus, the effect of N deprivation on GCN2 function might be indirect, by limiting the cellular uptake of EAA such as R. Consistently, GCN2 mutants were highly sensitive to dietary R deprivation. Through its role as exchange factor, N has been shown to affect mTORC1 activity and protein translation (Krall et al., 2016). Our results indicate that in addition to mTORC1, N deprivation might also activate GCN2 *in vivo*. Interestingly, asparagine synthetase (ASNS) is a direct target gene of ATF4 in mammals (Pathria et al., 2019). Furthermore, pharmacological inhibition of GCN2 sensitizes cancer cells with low expression levels of ASNS to the antileukemic agent 1-asparaginase (Nakamura et al., 2018), suggesting that the effect of N deprivation on GCN2 function are conserved from flies to mammals.

Our study also highlights that full amino acid starvation poses different stress from individual EAA deprivation, and is dealt with by a GCN2 independent mechanism. Consistently, data from HEK293 cells suggest that protein synthesis under full amino acid starvation is controlled by phosphorylation of eIF2B and is independent of changes in GCN2/eIF2α phosphorylation (Wang and Proud, 2008). Furthermore, in HepG2 cells total amino acid starvation decreases 4E-BP phosphorylation, suggesting that full amino acid starvation might involve the TOR pathway (Palii et al., 2009). However, more *in vivo* work is needed to understand the mechanisms by which cells sense and response to single and total amino acid starvation.

GCN2 kinase coordinates amino acid starvation stress response by activating the key transcription factor ATF4 (Harding et al., 2003). Here, we showed that increased *Atf4* expression is necessary but not sufficient to compensate for GCN2 mediated functions under deprivation of EAA and that the rescue efficiency of *Atf4* over-expression depends on which EAA is being deprived in the diet. Furthermore, transcriptomic profiling showed that ATF4 target genes were not significantly enriched among GCN2-dependent genes under R deprivation, consistent with the finding that overexpression of ATF4 only caused a slight rescue in starvation sensitivity of GCN2 mutants. One explanation for our observations is that GCN2 induces *Atf4* translation to different degrees in response to starvation of different EAA, as valine deprivation induced the highest ATF4 protein levels whereas deprivation of isoleucine resulted in very weak induction of ATF4 protein levels (Palii et al., 2009). Interestingly, I restriction, but not L restriction improved metabolic health in mice (Yu et al., 2021) and blood levels of I correlated with increased mortality in humans, while blood levels of L correlated with decreased mortality (Deelen et al., 2019), suggesting that these EAA have different physiological functions and are regulated by different upstream pathways. Certain EAA may trigger distinct transcription factors and co-activators, which may act in concert with ATF4 to fully facilitate its function, as several studies have documented that ATF4 and other transcription factors including ATF2, ATF3, ATF5, and cJUN interact together to regulate amino acid stress response (Bruhat et al., 2009; Kilberg et al., 2012). Moreover, we observed no rescue of GCN2-dependent functions upon *Atf4* over-expression in *Gcn2*-knockout flies when several EAA were individually deprived from the diet, suggesting that under these conditions GCN2 may activate transcription factor(s) other than ATF4. It has been shown that GCN2 activates nuclear-factor κB (NF-κB), *via* phosphorylation of eIF2α in mouse embryonic fibroblasts (Jiang et al., 2003). Thus, although it is well established that ATF4 is a primary component of amino acid stress response, our data suggest that, depending on which EAA is being deprived, GCN2 might activate additional transcription factors that work alone or in concert with ATF4 to regulate amino acid stress response. Therefore, for an accurate understanding of the role of ATF4 in amino acid stress response, the GCN2/ATF4 pathway should be studied in response to deprivation of individual amino acids. The mechanism by which deprivation of different amino acids selectively triggers ATF4 and the additional transcription factors are presently unknown.

## Data Availability

The data are now publicly available and can be accessed *via* the following link: https://www.ncbi.nlm.nih.gov/geo/query/acc.cgi?acc=GSE203210.

## Supplementary Material

Supplementary Figure S1 Generation and characterization of *Drosophila Gcn2* null mutants. **(A)** Schematic representation of the Drosophila *Gcn2* gene locus. *Gcn2* null mutants were generated *via* homologous recombination by replacing the complete *Gcn2* open reading frame with a *white* ^*hs*^ marker gene. 5′ and 3′ homologous arms used for homologous recombination are indicated as grey bars. Coding exons are indicated as black/blue bars, non-coding exons as white bars. **(B)** PCR on genomic DNA confirmed absence of *Gcn2*-specific PCR products in *Gcn2* ^*1*^ mutant flies. Genomic positions of PCR fragments are indicated in **(A)**. **(C)** Northern blot analysis of *Gcn2* expression in *w* ^*Dah*^ control and *Gcn2* ^*1*^ mutant flies. *RpL32* was used as the loading control. Expression of neighbouring genes *CG11337* and *CG31002* was unchanged in *Gcn2* ^*1*^ mutants. **(D)** Immunoblotting analysis of eIF2a phosphorylation (p-eIF2a) in *Gcn2* ^*1*^ mutants. p-eIF2a was normalised to total-eIF2a (t-eIF2a) levels. *n* = 3 biologically independent samples. Data are presented as mean ± s.e.m. Unpaired, two-tailed t-test. **(E,F)** Egg-to-adult viability **(E)** and body weight **(F)** of *w* ^*Dah*^ control and *Gcn2* ^*1*^ mutant flies on standard SYA diet. *n* = 10 vials with 25 embryos per vial **(E)** and *n* = 10 pairs of flies (females: *Gcn2* ^*1*^; males: *w* ^*Dah*^ and *Gcn2* ^*1*^), *n* = 9 pairs of flies (females: *w* ^*Dah*^) **(F)**. Median, 25th and 75th percentiles, and Tukey whiskers are indicated. Unpaired, two-tailed t-test.

Supplementary Figure S2 GCN2 function is essential for survival under deprivation of arginine but not methionine in males. **(A)** Cumulative egg laying of *w* ^*Dah*^ control and *Gcn2* ^*1*^ males under deprivation of individual EAA. *n* = 10 vials each condition of each genotype. Two-tailed, Mann-Whitney test. Median, 25th and 75th percentiles, and Tukey whiskers are indicated. **(B–D)** Survival of *w* ^*Dah*^ control and *Gcn2* ^*1*^ males under full deprivation of arginine **(B)**, methionine **(C)**, and all amino acids **(D)**. *Gcn2* ^*1*^ males had a significantly increased lifespan on Yaa compared to *w* ^*Dah*^ males. *n* = 150 flies per genotype, *p* = 0.04, log-rank test. **(B)**, *Gcn2* ^*1*^ males were significantly shorter-lived than *w* ^*Dah*^ males upon R deprivation (*n* = 200 flies, *p* = 2.1 × 10^−37^, log-rank test). **(C)**, *Gcn2* ^*1*^ mutant males were significantly longer-lived than *w* ^*Dah*^ males under M restriction (*n* = 200 flies, *p* = 1.2 × 10^−18^, log-rank test). **(D)**, *Gcn2* ^*1*^ mutant and *w* ^*Dah*^ control males showed comparable survival upon restriction of all amino acids (*n* = 180 flies for *w* ^*Dah*^, *n* = 200 flies for *Gcn2* ^*1*^, *p* = 0.72, log-rank test). Survival curves of *w* ^*Dah*^ and *Gcn2* ^*1*^ males on the Yaa diet are the same in **(B–D)**.

Supplementary Figure S3 GCN2 function is essential for feeding behaviour under long-term deprivation of asparagine. **(A–E)** Proboscis extension assay of *w* ^*Dah*^ and *Gcn2* ^*1*^ females after long-term (day 4) deprivation of NEAA. **(A)** Feeding rates of *Gcn2* ^*1*^ mutants were significantly reduced upon deprivation of all EAA and NEAA. *n* = 8 vials with 5 flies per vial. **(B–D)** Feeding behaviour of *w* ^*Dah*^ control and *Gcn2* ^*1*^ mutants was not affected by deprivation of C, E, Q, and Y **(B)**, D, S, and P **(C)** or A and G **(D)**. Feeding rates of *Gcn2* ^*1*^ mutants were reduced upon N deprivation only **(C)**. **(B–D)** *n* = 10 vials with 5 flies per vial. **(E)** Reduced feeding of *Gcn2* ^*1*^ mutants induced by NEAA deprivation was rescued by supplementation with N (EAA + N). *n* = 10 vials with 5 flies per vial. **(F)** Blue dye feeding assay on representative EAA deficient diets. *n* = 6 replicates with 5 flies per replicate. Data are present as mean ± s.e.m. One-way ANOVA followed by Dunnett’s multiple comparisons test, *p* values were adjusted for multiple comparisons.

Supplementary Figure S4 GCN2 mutants showed decreased starvation resistance under long-term deprivation of asparagine. **(A)** Total protein content per fly of *w* ^*Dah*^ and *Gcn2* ^*1*^ females upon deprivation of individual EAA. *n* = 4 replicates with 5 females per replicate. The same flies corresponding to the TAG measurements in Figure 4A were used for protein determination. Data are present as mean ± s.e.m. One-way ANOVA followed by Dunnett’s multiple comparisons test, *p* values were adjusted for multiple comparisons. **(B,C)** Survival under full starvation of *w* ^*Dah*^ and *Gcn2* ^*1*^ females. **(B)** Flies were fed for seven days with -All, -NEAA, and -EAA Yaa diets, followed by full starvation. *w* ^*Dah*^ control flies preconditioned with diets deprived of EAA and NEAA showed a strong increase in survival compared to the Yaa diet: -NEAA (*p* = 2.8 × 10^−16^), -EAA (*p* = 3.0 × 10^−12^, log-rank test), while only a mild increase was observed upon deprivation of -All (*p* = 1.2 × 10^−3^ , log-rank test). *Gcn2* ^*1*^ mutants preconditioned on -All (*p* = 6.0 × 10^−8^), -NEAA (*p* = 2.4 × 10^−3^), -EAA (*p* = 3.9 × 10^−9^, log-rank test) showed significantly decreased starvation resistance compared to the Yaa diet. **(C)** Survival under full starvation of females preconditioned on individual NEAA-deficient diets. Only deprivation of N significantly increased survival of *w* ^*Dah*^ control flies compared to the Yaa diet: -G (*p* = 0.83), -A (*p* = 0.38), -D (*p* = 0.35), -S (*p* = 0.65), -P (*p* = 0.21), -N (*p* = 3.6 × 10^−6^, log-rank test). *Gcn2* ^*1*^ mutants were significantly short-lived only upon N deprivation: -G (*p* = 0.88), -A (*p* = 0.44), -D (*p* = 0.42), -S (*p* = 0.15), -P (*p* = 0.69), -N (*p* = 1.7 × 10^−5^), log-rank test. *n* = 100 flies for each diet and genotype.

Supplementary Figure S5 Transcriptional profiling identifies diet and GCN2 dependent changes. **(A)** Venn diagram representation of the overlap of upregulated and downregulated gene sets between *w* ^*Dah*^ controls and the *Gcn2* ^*1*^ mutants under each diet. Overlaps were significant for all diets: upregulated: -R, *p* = 1.8 × 10^−148^, -M, *p* = 0, -All, *p* = 0; downregulated: -R, *p* = 1.5 × 10^−38^, -M, *p* = 6.8 × 10^−26^, -All, *p* = 3.9 × 10^−153^, Fisher’s exact test. **(B)** Venn diagram representation of differentially expressed genes of *w* ^*Dah*^ controls and *Gcn2* ^*1*^ mutants overlapping with ATF4-target genes. Overlaps were significant for all diets: *w* ^*Dah*^ controls: -R, *p* = 0.005, -M, *p* = 0.02, -All, *p* = 0.06; *GCN2* ^*1*^ mutants: -R, *p* = 0.04, -M, *p* = 0.02, -All, *p* = 0.06, Fisher’s exact test. **(C)** Venn diagram representation of GCN2 dependent genes overlapping with ATF4-target genes. No significant overlap was detected by Fisher’s exact test. **(D)** Expression level of ASNS in *w* ^*Dah*^ controls and *Gcn2* ^*1*^ mutants on each diet based on the RNA Seq analysis. *n* = 3 replicates each genotype per diet. Data are present as mean ± s.e.m. Two-way ANOVA followed by Bonferroni’s post-hoc test, *p* values were adjusted for multiple comparisons.

Supplementary Figure S6 Over-expression of *Atf4* partially rescued starvation sensitivity of *Gcn2* ^*1*^ mutants in an amino acid dependent manner. **(A–J)** Survival of flies preconditioned on individual EAA deficient diets for seven followed by full starvation. Ubiquitous adult-specific overexpression of *Atf4* in *Gcn2* ^*1*^ mutants partially rescued starvation sensitivity dependent on EAA deficiency: -L: *p*_*da-GS*_ = 2.1 × 10^−38^, *p*_*UAS-Atf4*_ = 4.8 × 10^−15^ **(A)**, -W: *p*_*da-GS*_ = 2.4 × 10^−43^, *p*_*UAS-Atf4*_ = 9.0 × 10^−36^ **(B)**, -V: *p*_*da-GS*_ = 4.3 × 10^−18^, *p*_UAS*-*Atf4_ = 3.4 × 10^−13^ **(C)**, -T: *p*_*da-GS*_ = 5.8 × 10^−20^, *p*_*UAS-Atf4*_ = 1.1 × 10^−22^ **(D)**, -K: *p*_*da-GS*_ = 5.6 × 10^−15^, *p*_*UAS-Atf4*_ = 6.6 × 10^−6^ **(E)**, -R: *p*_*da-GS*_ = 1.3 × 10^−5^, *p*_*UAS-Atf4*_ = 3.4 × 10^−5^ **(F)**, -I: *p*_*da-GS*_ = 8.3 × 10^−5^, *p*_*UAS-Atf4*_ = 0.04 **(G)**, -F: *p*_*da-GS*_ = 7.9 × 10^−15^, *p*_*UAS-Atf4*_ = 0.19 **(H)**, -H: *p*_*da-GS*_ = 2.3 × 10^−5^ , *p*_*UAS-Atf4*_ = 0.79 **(I)**, -M: *p*_*da-GS*_ = 0.01, *p*_*UAS-Atf4*_ = 0.28 **(J)** log-rank test. *Atf4* over-expression in **(A–J)** was induced by 100 μM RU486 using the *da-GS* driver. *n* = 100 flies for each condition of each genotype.
